# Supplementary material for: In-plane uniaxial pressure-induced out-of-plane antiferromagnetic moment and critical fluctuations in BaFe2As2
Source: Nat Commun. 2020 Nov 12;11:5728. doi: 10.1038/s41467-020-19421-5 (PMC7665052; doi:10.1038/s41467-020-19421-5)
Supplement: Supplementary file 1 — Supplementary Information [file 41467_2020_19421_MOESM1_ESM.pdf]

Supplemental Information: In-plane uniaxial pressure-induced out-of-plane antiferromagnetic moment and critical fluctuations in $BaFe_2As_2$

Sample and uniaxial-pressure device

Figure S1 shows the sample set used for our polarized neutron scattering experiments. 12 pieces of large, flat $BaFe_2As_2$ single crystals were carefully selected and cut into rectangular shape along their tetragonal $[1, 1, 0]$ directions. The well-cut single crystals were inserted into the uniaxial-pressure device as shown in Fig. S1. The uniaxial pressure was applied by a specially designed aluminum spring washer pressed by an aluminum screw. The pressure were estimated to be $P \approx 20 \pm 5$ MPa. The device can be mounted onto the sample stick with two configurations [Fig. S1(a) and (b)], corresponding to the scattering planes of $[H, 0, L]$ and $[0, K, L]$, respectively.

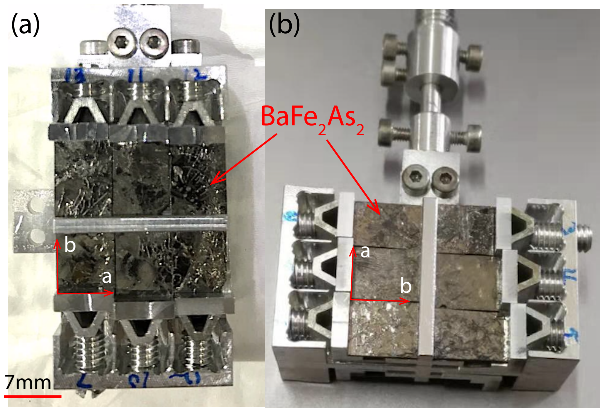

FIG. S1: $BaFe_2As_2$ sample set for neutron scattering. The scattering planes in (a) and (b) are $[H, 0, L]$ and $[0, K, L]$, respectively, as the shorter orthorhombic b axis favors the direction along which the uniaxial pressure is applied.

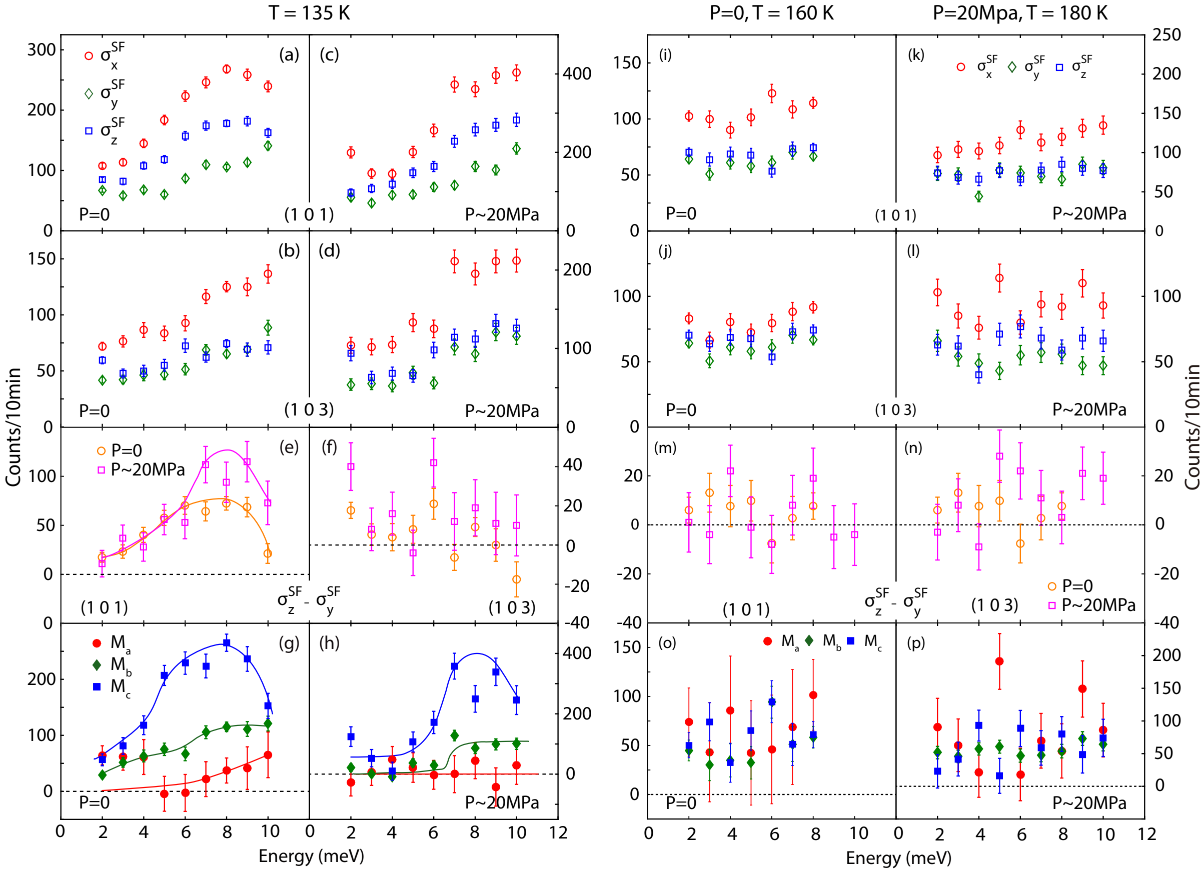

FIG. S2: (a-d) Energy scans of $\sigma_x^{\rm SF}$ (red circle), $\sigma_y^{\rm SF}$ (green diamond), and $\sigma_z^{\rm SF}$ (blue square) under (a, b) $P = 0$ and (c, d) 20 MPa at the two antiferromagnetic wave vectors $\mathbf{Q}_1 = (1, 0, 1)$ and $\mathbf{Q}_2 = (1, 0, 3)$, measured at $T = 135$ K $< T_N$. (e, f) Energy dependent $\sigma_z^{\rm SF} - \sigma_y^{\rm SF}$ measured under $P = 0$ and 20MPa at $\mathbf{Q}_1 = (1, 0, 1)$ and $\mathbf{Q}_2 = (1, 0, 3)$. (g, h) Energy dependence of M_a, M_b, and M_c at 135 K extracted from the raw data in (a-d) following the relations as shown in Fig. 1 of main text. The solid lines are guides to the eyes. (i, j) Identical scans as (a, b) but at $T = 160$ K and $P = 0$, (k, l) Identical scans as (c, d) but at $T = 180$ K and $P \sim 20$ MPa. (m, n) $\sigma_z^{\rm SF} - \sigma_y^{\rm SF}$ as a function of energy transfer measured under $P = 0$ ($T = 160$ K) and 20 MPa ($T = 180$ K) at $\mathbf{Q}_1 = (1, 0, 1)$ and $\mathbf{Q}_2 = (1, 0, 3)$. (o, p) $M_{a,b,c}$ calculated from the data shown in (i)-(l).

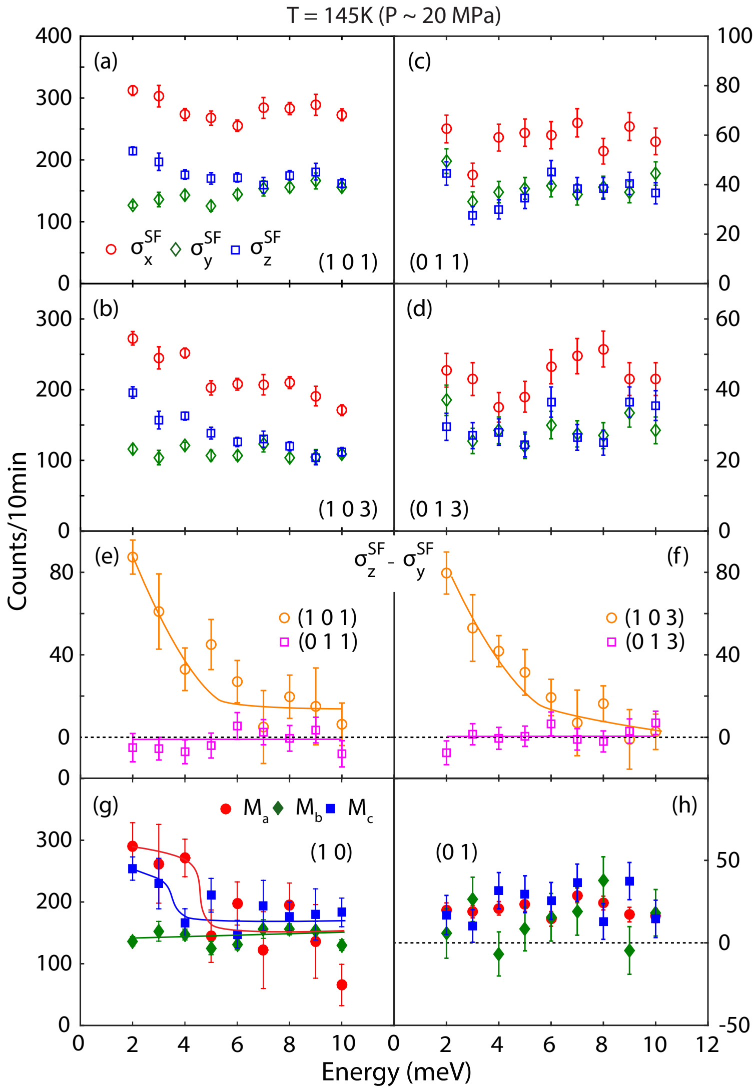

FIG. S3: Energy scans of $\sigma_x^{\rm SF}$, $\sigma_y^{\rm SF}$ and $\sigma_z^{\rm SF}$ under $P \sim 20$MPa at (a, b) $\mathbf{Q} = (1, 0, L)$ and (c, d) $(0, 1, L)$ $(L = 1, 3)$, measured at $T = 145$ K slightly above T_N. (e, f) Energy dependence of $\sigma_z^{\rm SF} - \sigma_y^{\rm SF}$ measured at (e) $(1, 0, 1)$ and $(0, 1, 1)$ and (f) $(1, 0, 3)$ and $(0, 1, 3)$. (g, h) Energy dependence of M_a, M_b, and M_c extracted from the raw data in (a-d). The solid lines are guides to the eyes.

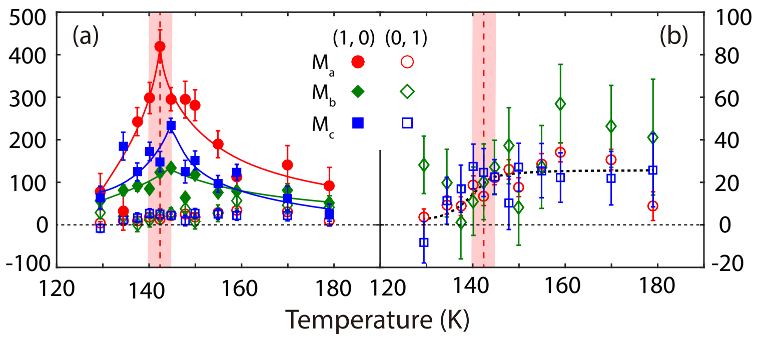

FIG. S4: (a) Temperature dependence of M_a, M_b, and M_c obtained from spin-flip scattering $\sigma_{\alpha}^{SF}(\alpha = x, y, z)$ measured at $\mathbf{Q} = (1, 0, L)$ (solid symbols) and $(0, 1, L)(L = 1, 3)$ (open symbols). (b) Zoomed-in view of the M_a, M_b, and M_c for $\mathbf{Q} = (0, 1, L)$. Vertical dashed line and pink area mark the magnetic transition T_N. The solid lines and black dashed lines are guides to the eyes.

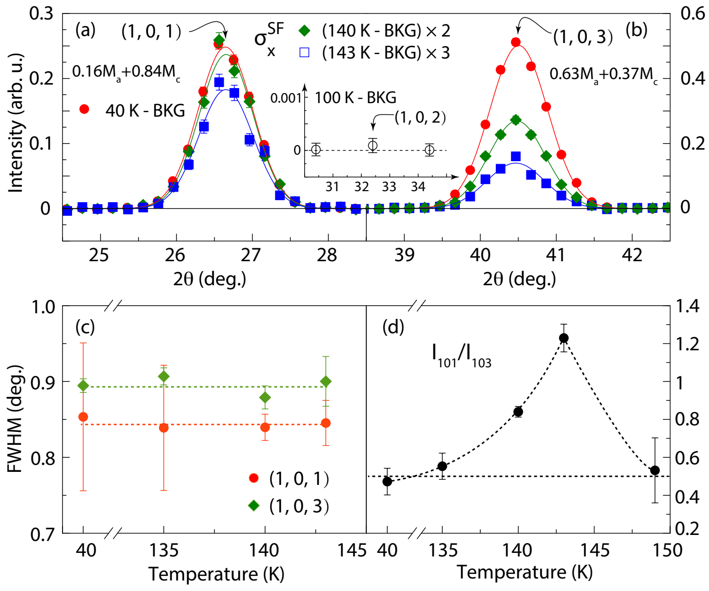

FIG. S5: Elastic $\theta/2\theta$ scans of σ_x^{SF} across (a) $(1, 0, 1)$ and (b) $(1, 0, 3)$ at various temperatures. Similar scans of σ_z^{SF} are discussed in Fig. 4 of the main text. (c) FWHM of the elastic $(1, 0, 1)$ and $(1, 0, 3)$ scans across T_N/T_s. (d) Temperature dependence of I_{101}/I_{103}.
